# A new glycation product ‘norpronyl-lysine,’ and direct characterization of cross linking and other glycation adducts: NMR of model compounds and collagen

**DOI:** 10.1042/BSR20130135

**Published:** 2014-03-14

**Authors:** Peter T. B. Bullock, David G. Reid, W. Ying Chow, Wendy P. W. Lau, Melinda J. Duer

**Affiliations:** *Department of Chemistry, University of Cambridge, Lensfield Road, Cambridge CB2 1EW, U.K.

**Keywords:** ADP-ribose, advanced glycation endproducts, Maillard reaction, ribose, ribose-5-phosphate, solid-state NMR, ADPR, ADP-ribose, AGE, advanced glycation endproduct, CEL, carboxyethyl lysine, CML, carboxymethyl lysine, CP, cross polarization, DOPDIC, N6-{2-{[(4S)-4-ammonio-5-oxido-5-oxopentyl]amino}-5-[(2S)-2,3-ydihydroxypropyl]-3,5-dihydro-4H-imidazol-4-ylidene}-L-lysinate, GOLD, glyoxal lysine dimer, MOLD, methylglyoxal lysine dimer, PAR, poly-ADP-ribose, PLL, poly-L-lysine, POST-C7, permutationally offset stabilized C7, R5P, ribose-5-phosphate, SQ–DQ, single quantum–double quantum, ssNMR, solid-state NMR

## Abstract

NMR is ideal for characterizing non-enzymatic protein glycation, including AGEs (advanced glycation endproducts) underlying tissue pathologies in diabetes and ageing. Ribose, R5P (ribose-5-phosphate) and ADPR (ADP-ribose), could be significant and underinvestigated biological glycating agents especially in chronic inflammation. Using [U-^13^C]ribose we have identified a novel glycoxidation adduct, 5-deoxy-5-desmethylpronyl-lysine, ‘norpronyl-lysine’, as well as numerous free ketones, acids and amino group reaction products. Glycation by R5P and ADPR proceeds rapidly with R5P generating a brown precipitate with PLL (poly-L-lysine) within hours. ssNMR (solid-state NMR) ^13^C–^13^C COSY identifies several crosslinking adducts such as the newly identified norpronyl-lysine, *in situ*, from the glycating reaction of ^13^C_5_-ribose with collagen. The same adducts are also identifiable after reaction of collagen with R5P. We also demonstrate for the first time bio-amine (spermidine, N-acetyl lysine, PLL) catalysed ribose 2-epimerization to arabinose at physiological pH. This work raises the prospect of advancing understanding of the mechanisms and consequences of glycation in actual tissues, *in vitro* or even *ex vivo*, using NMR isotope-labelled glycating agents, without analyses requiring chemical or enzymatic degradations, or prior assumptions about glycation products.

## Abstract 2

NMR reveals numerous early and advanced glycation products, including a newly recognized ‘norpronyl-lysine,’ and cross links in solution, intact collagen and model systems. Solid state methods are directly applicable to *in vitro* and *in vivo* glycation pathway and product characterization.

## INTRODUCTION

Glycation (non-enzymatic glycosylation) is the reaction of sugars to form covalent adducts with proteins. It occurs wherever sugars and proteins are in contact, usually initially through so-called Maillard reactions (see Supplementary Data Section S1; available at http://www.bioscirep.org/bsr/034/bsr034e096add.htm for summaries of these, and other glycation reactions and products and their abbreviations). Initial products react further to form AGEs (advanced glycation endproducts), including protein modifications, sugar degradation products and protein crosslinks, the latter implicated in numerous conditions including diabetes, atherosclerosis, osteoarthritis and cataracts [1,2]. The Maillard reaction with glucose *in vivo* is slow at physiological temperature and pH due to its low reactivity, so only long-lived structural proteins such as collagen and elastin are significantly affected by crosslinking arising from glycation by this sugar [2].

Collagen crosslinking is a poorly understood pathological consequence of glycation, leading to irreversible degeneration of mechanical properties, such as stiffer, more brittle connective tissue fibres. Both crosslinking and simple modifications of amino acid side-chains can lead to abnormal biochemical functionality of collagen by changing the charge profile of the collagen fibrils, with unpredictable consequences [3]. For instance, modification of lysine residues to the relatively common AGEs *N^ε^*-CML [(carboxymethyl)lysine] and *N^ε^*-CEL [(carboxyethyl)lysine] changes a positively charged into a negatively charged side-chain.

Research has focused on glycation by glucose, assumed to be the most relevant sugar in both non-pathological tissues and hyperglycaemic conditions. However, glucose is not an efficient glycating agent as it overwhelmingly adopts its thermodynamically stable pyranose form, rather than the reactive open-chain aldehyde form. Pentoses such as ribose and especially R5P (ribose-5-phosphate) are much more potent glycating agents, R5P reacting with amines 150-fold faster than glucose [4,5]. R5P and the mechanistically almost indistinguishable ADPR (adenosine diphosphate ribose) are both released into the extra-cellular matrix on cell necrosis, so are present in chronic inflammation where there is long-term cellular damage, and so could be potent biologically significant and, to date, under-investigated glycating agents. Some established reaction pathways to AGEs are not available to R5P or ADPR due to phosphoesterification of the terminal ribosyl 5-carbon; equally, it is possible that there are glycation products of R5P or ADPR that do not occur for glucose. The aim of this work is to identify the possible glycation products of ribose, R5P and ADPR in both model systems and collagen itself.

Glycation products can be detected by a variety of techniques in different tissues and experimental models systems [6–20], including solution state NMR (hereafter simply referred to as ‘NMR’ in distinction to ssNMR (solid-state NMR)), which can detect soluble AGEs [12,21–23]. Although all these previous approaches yield valuable chemical and mechanistic insights, they are only applicable with considerable prior sample preparation to the large insoluble structural proteins, which are significant pathogenic targets of tissue glycation. ssNMR, however, bypasses many of these difficulties and can access a wealth of chemical information from these solid materials in a close to native state. Thus, in this work we develop NMR spectroscopic and labelling approaches to analysing mixtures of glycation products, in particular those from ribose, R5P and ADPR, and to exploring the products of glycation of collagen itself. The approaches we develop could find applications in the study of tissue glycation in hyperglycaemic diseases such as diabetes.

## MATERIALS AND METHODS

Reagents were from Sigma–Aldrich except [U-^13^C]ribose, which was from Cambridge Isotope Laboratories. PLL (poly-L-lysine) hydrobromide was molecular mass 4,000–15,000 Da. Reagent salt forms were: Spermidine trihydrochloride, disodium R5P, sodium ADPR. Dry Type I collagen (bovine Achilles tendon) was rehydrated by soaking overnight in aqueous acetic acid (10%), homogenized with a hand held plunger homogenizer, and washed with distilled water and centrifuged repetitively until no longer acidic, and stored wet at 4°C until used. All incubations were performed at 37°C in 50 mM potassium phosphate at pH 7.4 in 5% D_2_O/H_2_O to which a few crystals of sodium azide had been added to ensure sterility, unless otherwise indicated, and progress was monitored periodically by NMR. Glycation products were shown to be reproducible by repeating each experiment.

### Glycation of model amines

Composition of individual reaction mixtures in 1.05 ml (unless stated otherwise) solution are summarized below. To facilitate periodic analyses by NMR these reactions were performed in 4 mm outer diameter glass NMR tubes.

#### α-N-acetyl lysine

AcLys 9.5 mg, ribose 7.5 mg; AcLys 9.5 mg, [U-^13^C]ribose 7.5 mg; AcLys 9.5 mg R5P 13 mg.

#### Spermidine

Spermidine 13 mg, ribose 7.5 mg; Spermidine 13 mg, R5P 13 mg.

#### PLL

PLL 12 mg, ribose 7.5 mg; PLL 12 mg, [U-^13^C]ribose 7.5 mg; PLL 12 mg, ADPR 28 mg.

#### Controls

Ribose (7.5 mg), [U-^13^C]ribose (7.5 mg), and R5P (12 mg), were each incubated for a month; the spectrum of each was unchanged after this time.

### Glycation of samples for ssNMR

#### Large scale glycation of PLL

550 mg PLL and 750 mg R5P were incubated in 45 cm^3^ buffer containing sodium azide for a day. The resultant brown precipitate was washed with 40 ml water and centrifuged, three times, and air dried overnight for ssNMR.

#### Collagen incubations under biomimetic conditions

2 g wet mass of collagen was used in all incubations. A few crystals of sodium azide were added to incubations for sterility. All glycated solids were washed with 40 ml water and centrifuged, three times, and air dried overnight for ssNMR.

Collagen and R5P: a mixture of collagen and 60 mg R5P disodium salt was made up to 4 cm^3^ with water and incubated at 310 K for 14 days. Phosphate buffer (1 M, 2 ml) was added after 8 days. The glycated collagen was distinctly yellow at this point.

Collagen and [U-^13^C]ribose: a mixture of collagen and [U-^13^C]ribose (35 mg) was made up to 4 cm^3^ with phosphate buffer (50 mM) and incubated for 69 days, at which point the collagen was distinctly orange. The pale yellow supernatant was collected for NMR analysis.

Collagen and R5P (pH 9, sonicated): a mixture of collagen (2 g, wet) and R5P (60 mg) was made up to 4 cm^3^ with phosphate buffer (100 mM, pH 9.0 attained with NaOH), sonicated and incubated at 310 K for 11 days, at which point the collagen was yellow.

### NMR

All ^13^C and ^31^P experiments were performed in H_2_O/^2^H_2_O using standard methodology on a Bruker 11.8 Tesla Avance-500 standard bore spectrometer, at frequencies of 500.1 MHz (^1^H), 125.6 MHz (^13^C) and 202 MHz (^31^P). Pulse-acquire and ^13^C-^13^C COSY, was performed with continuous broadband decoupling. ^13^C and ^31^P chemical shifts were referenced, respectively, to internal TSP (trimethylsilapentane) sulfonate sodium salt, and external 85% phosphoric acid, at 0 ppm.

### ssNMR

All measurements were performed on a Bruker 9.4 T Avance-400 wide bore spectrometer, with a standard Bruker MAS 4 mm double resonance probe operating at 400.4 MHz, 100.6 MHz and 162.1 MHz for ^1^H, ^13^C and ^31^P, respectively. Samples were in 4 mm zirconia rotors and spun at 12.5 kHz at the ‘magic’ angle of 54.7° unless stated otherwise. CP (cross-polarization) from ^1^H was used to enhance signal intensity (^1^H π/2 pulse 2.5 μs, CP field strength 70 kHz, CP time 2.5 ms, spinal64 broadband decoupling (100 kHz field) during acquisition, repetition time 2 s. SQ–DQ (single quantum–double quantum) ^13^C-^13^C ssNMR correlation was achieved using POST-C7 (permutationally offset stabilized C7) [24] methodology and MAS (magic angle spinning) frequency of 10 kHz. ^13^C and ^31^P spectra were referenced to the glycine Cα signal at 43.1 ppm, and to hydroxyapatite at 2.6 ppm, respectively.

## RESULTS

Many protein gyrations occur through lysyl amines, so in this work these processes were modelled by using the simpler amines PLL, AcLys and spermidine as models before examining the glycation of collagen. Glycation reactions with ribose, R5P and ADPR all produced numerous AGEs and AGE analogues, which we categorize as (i) sugar modifications, (ii) protein side chain modifications and (iii) cross links.

### Sugar modifications

The majority of Maillard reactions do not in fact consume the amine [5], rather the amine catalyses sugar degradations and rearrangements, which would not occur in the absence of amines.

#### Catalytic epimerization of ribose

**Scheme 1:**
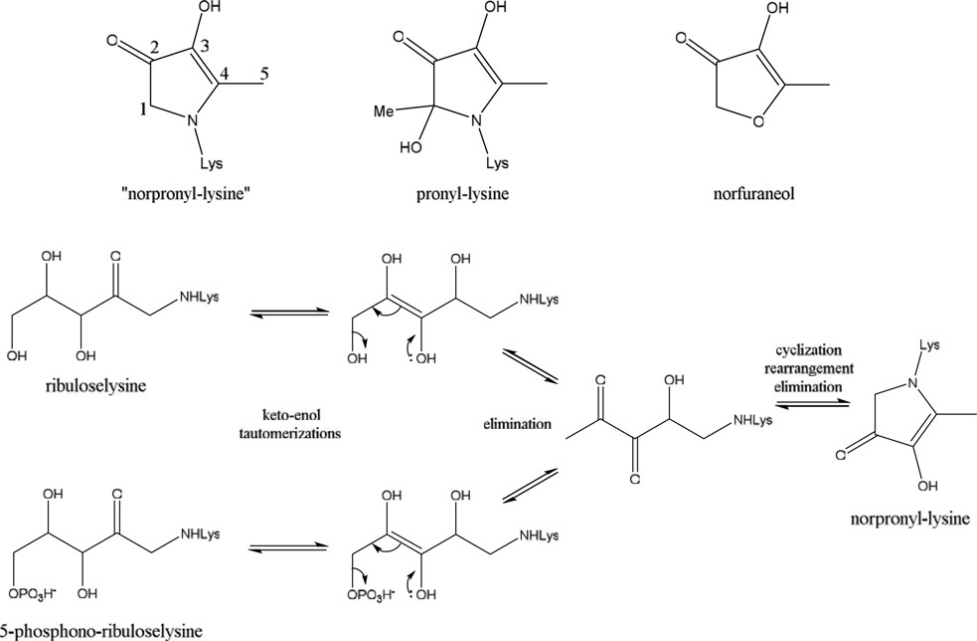
Postulated mechanism of formation of norpronyl-lysine from the Amadori products of both ribose and R5P with lysine Note the similarity to the established mechanism of formation of norfuraneol in the cyclization, rearrangement and elimination of water (Online Supplemental Material Section S1 Scheme S2).

All the amines catalyse epimerization of ribose into arabinose, readily shown by the replacement of ribose NMR signals by signals characteristic [25] of the two anomeric forms of arabinose as the reactions proceed (Supplementary Data Section S2 Figure S2; available at http://www.bioscirep.org/bsr/034/bsr034e096add.htm). This result is significant because, although previously observed in strong alkali [26], such rearrangements have not been reported before in such mild biomimetic conditions as those used in this work. This suggests that the amine is integral to the mechanism of epimerization, with the reaction mechanism in Supplementary Data Section S1 Scheme 1 explaining the observation if the Amadori rearrangement (Step 2) is significantly reversible.

#### Generation of carboxylic acids

Simple carboxylic acids were common Maillard products detected in all reaction systems. Supplementary Data Section S3 Table S1 (available at http://www.bioscirep.org/bsr/034/bsr034e096add.htm) summarizes likely assignment of ^13^C solution-state NMR signals from possible 1- and 2-carbon atom acids resulting from oxidative degradation of ribose and R5P. Formic, acetic and glycolic acids were abundant AGEs from all reaction mixtures, with oxalic acid also common although generated in smaller quantities; the occurrences of these acids is summarized in Supplementary Data Section 3 Table S2. Acetic and glycolic acids are unlikely to have any pathological effects, formic acid is metabolized rapidly, but even so its generation by glycation in the eyes may have pathological consequences due to its high ocular toxicity. Oxalic acid generation in sufficient concentrations may lead to pathological precipitation of calcium oxalate, the most significant component of kidney stones.

#### Norfuraneol

This was observed in all ribose/amine incubations, but not in R5P/amine incubations. A full ^13^C solution-state NMR assignment of ^13^C norfuraneol is given in Supplementary Data Section S4 Table S3 (available at http://www.bioscirep.org/bsr/034/bsr034e096add.htm). The lack of norfuraneol as an AGE in reactions of R5P was expected [5] as R5P lacks the C5 hydroxyl group necessary for the final cyclization step that produces norfuraneol (see Online Supplemental Material Scheme S2).

### Protein side chain modifications

#### Amadori compound

All incubations of ribose or R5P with amines yielded a small transient ^13^C NMR signal at 208–210 ppm (Online Supplementary Material Section S5; available at http://www.bioscirep.org/bsr/034/bsr034e096add.htm), most likely due to the Amadori product, ribuloselysine (or 5-phospho-ribuloselysine, see Supplementary Data Scheme S1), which is predicted to be present at low concentrations according to steady-state kinetics. The signal appears as a doublet of doublets (J=46 and 40 Hz) in the ^13^C_5_-ribose/AcLys system, and shows ^13^C-^13^C COSY correlations to signals at 57 and 79 ppm in the ^13^C_5_-ribose/PLL system, as expected for the ribuloselysine carbonyl carbon [27]. This suggests that the progress of the Maillard reaction *in vitro* can be monitored by the evolution of this ca. 209 ppm signal, since once it has disappeared, the initial glycation step (whereby free ribose becomes covalently bound to the amine) must be complete.

#### CML and CEL

The anticipated AGE, CML, was readily identified by ^13^C NMR spectroscopy as a pair of signals at 52.1 and 174.3 ppm (^13^C-enriched ribose experiments yielded two doublets, *J=* 53 Hz). In the liquid state this provides a very straightforward diagnostic for CML. However, since in a protein the peptide α-carbons and carbonyls resonate at ~50 and ~175 ppm, respectively, in the solid state there is no chance of resolving CML signals from those of the protein without selective ^13^C enrichment. No evidence was found for CEL as a glycation product with ribose, R5P or ADPR; since the methyl carbon of CEL gives a distinctive resonance at ~15 ppm [28], the absence of such a signal implies that very little CEL was generated.

#### Acetylation

^13^C-^13^C COSY analysis of ^13^C_5_-ribosylated PLL reveals a correlation between 23 and 177 ppm. This is characteristic of an acetyl amide, *N^ε^-*acetyl lysine, a known AGE.

#### Discovery of a novel pyrrolinone reductone AGE

**Figure 1:**
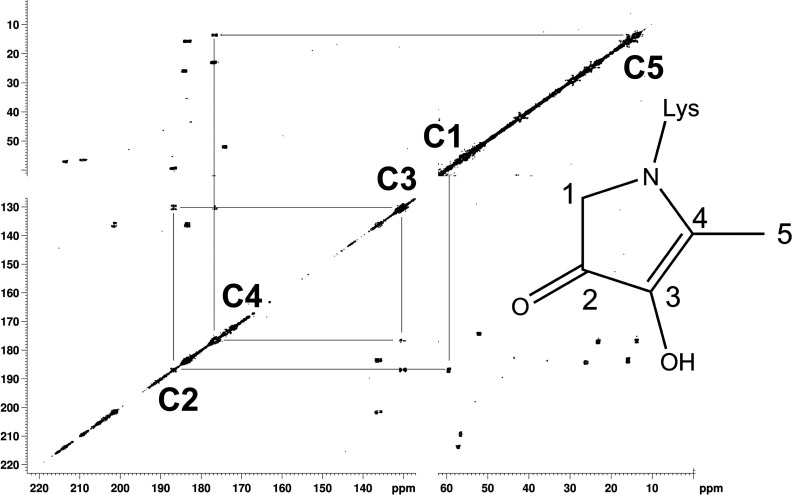
^13^C–^13^C COSY correlation spectrum of [U-^13^C]ribose glycated PLL Portions of the 2D ^13^C-^13^C COSY correlation spectrum acquired from PLL after 10 days incubation with ^13^C_5_-ribose. The data is presented as a contour plot in which the diagonal (bottom left to top right) represents a conventional 1D spectrum ‘viewed from above’. The off-diagonal ‘cross peak’ features connect signals between carbon atoms which are directly bonded to each other, at chemical shifts (i.e. frequencies) which are effectively Cartesian coordinates of the two bonded atoms. Cross peaks corresponding to the norpronyl group (shown) are assigned and connected by solid vertical and horizontal lines.

A set of five COSY correlated ^13^C resonances were well resolved in [U-^13^C]ribosylated PLL (Figure 1) and in [U-^13^C]ribosylated AcLys, at 59 ↔ 187 ↔ 130 ↔ 176 ↔ 14 ppm due to a previously unreported pyrrolinone reductone [29] AGE for which we propose the name ‘norpronyl-lysine’ for its similarity to the established AGE pronyl-lysine [30]. A full assignment of the ^13^C NMR spectrum of norpronyl-lysine including through-bond coupling constants (J_CC_ values) is given in Table 1.

**Table 1:** Full assignment of ^13^C_5_-norpronyl-lysine as observed after glycation of AcLys by [U-^13^C]ribose

carbon atom	Δ (ppm)	Multiplicity	*J* (Hz)
1	59.3	dd	43, 19
2	186.6	ddd	73, 43, 14
3	129.9	ddd	77, 73, 19
4	176.6	ddd	77, 44, 14
5	13.6	d	44

Norpronyl-lysine is postulated to form by 5-elimination from the Amadori product [31] followed by cyclization, rearrangement and dehydration (Scheme 1). This mechanism implies that R5P should undergo conversion to norpronyl-lysine more readily than ribose as elimination of a phosphate group proceeds preferably to elimination of hydroxide, and we do indeed observe norpronyl-lysine in the incubations of R5P with PLL and collagen (see later). In a similar experiment studying the reaction between R5P and AcLys in non-oxidative conditions, a compound by LC/MS of mass equivalent to [AcLys-NH_2_+96] was reported with no firm assignment [5], consistent with the generation of norpronyl-lysine, though unrecognized at the time. Reaction of PLL with R5P and ADPR results in rapid formation of a brown solid (see following section for more detailed discussion of the AGEs arising from these reactions) and thus we have to use ssNMR to assess the products from these glycation reactions. Amongst the many product signals (see following section) is one at 192 ppm, an unusual ^13^C chemical shift, higher in frequency than an acid, ester or amide but lower than a ketone, not reported before in glycation reactions with pentose sugars. By comparison with the ribose/PLL system it appears to be due to norpronyl-lysine. The change in chemical shift of the carbonyl group between liquid state and solid state (~5 ppm) is not unexpected, and is attributable to packing effects in solids which are not operative in the liquid, and/or changes in hydrogen bonding between solid and solution state.

The biological effects of norpronyl-lysine can only be speculated at, but the very similar lysine modification pronyl-lysine may be a free radical scavenging antioxidant which inhibits development of colonic precancerous lesions [32]. On the other hand, as a protein modification it is likely to have a negative impact on the functionality of collagen and on collagen recycling by collagenases.

### Crosslinking

#### PLL with R5P, and with ADPR

**Figure 2:**
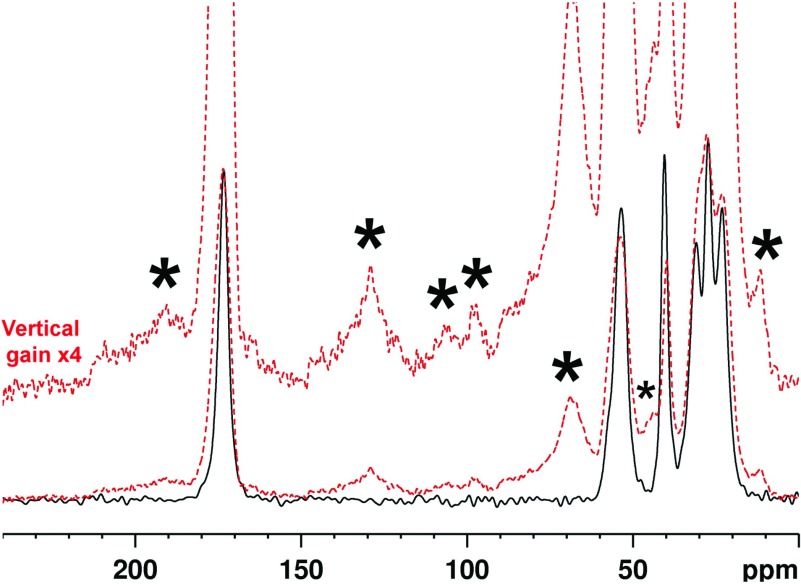
^13^C ssNMR from the PLL–R5P reaction ^13^C ssNMR of PLL before (black solid lines, bottom) and after (red, dashed lines) incubation with R5P. Glycation broadens the PLL peaks due probably to increased chemical diversity. The most significant new signals characterized by signal-to-noise ratios of greater than 5, are asterisked. Tentative assignments are: 130 and 10 ppm–MOLD; 130 ppm could indicate some GOLD; 70 ppm–secondary alcohol resonances from intact sugars or sugar degradation products bound into the polymer, and/or lysyl modifications such as N^ε^-glyceryl lysine; 44 ppm could correspond to C_ε_ of a modified lysine such as MOLD (which would explain the broadness as many modifications are possible). 192 ppm is from norpronyl-lysine. See Online Supplemental Material Section S1 Figure S1 for chemical structures of glycation products.

Reaction between R5P and PLL led to precipitation of a brown polymer after only a few hours, attributed to the formation of crosslinks between the strands of PLL. This dramatic glycation effect was also observed for ADPR and shown, by ^31^P and ^13^C NMR (Supplementary Data Section S6 Figures S4 and S5; available at http://www.bioscirep.org/bsr/034/bsr034e096add.htm, respectively), to proceed *via* elimination of ADP, consistent with the literature [5] and further corroborated by ^31^P ssNMR (Supplementary Data Section S6 Figure S6). Glycation of PLL by R5P led to generation of reaction products with ^13^C ssNMR signals consistent with the expected crosslinks MOLD (methylglyoxal lysine dimer) [33] and GOLD (glyoxal lysine dimmer) [34], with the resonance at 10 ppm particularly diagnostic of MOLD, as well as signal in the sugar region (60–80 ppm) from several overlapping components (Figure 2) consistent with both of these species.

### Glycation of collagen

#### Reactions with [U-13C]ribose

**Figure 3:**
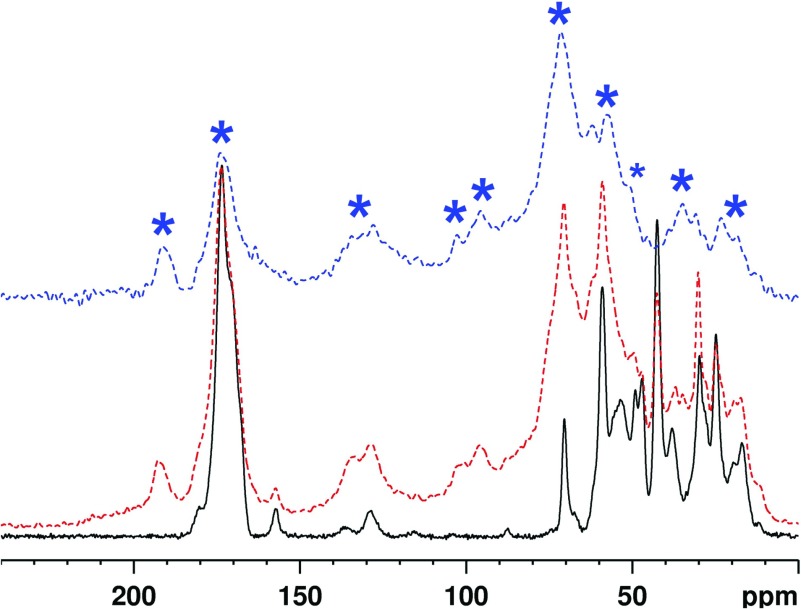
^13^C ssNMR of the collagen-[U-^13^C]ribose reaction ^13^C ssNMR spectrum of ^13^C_5_-ribosylated collagen (69 days incubation, red, dashed middle trace), and ^13^C 1D SQ–DQ spectrum ‘filtered’ using the POST-C7 technique (blue, dashed upper trace), shown relative to unglycated collagen incubated for the same period (black, lower trace). Resolved new signals derived from the ribose are marked with asterisks, but there are broad areas of new signal throughout the range 10–215 ppm as is evident from the 1D SQ–DQ filtered spectrum, which shows only signals from ^13^C directly bound to another ^13^C and hence unambiguously derived from [U-^13^C]ribose.

**Figure 4:**
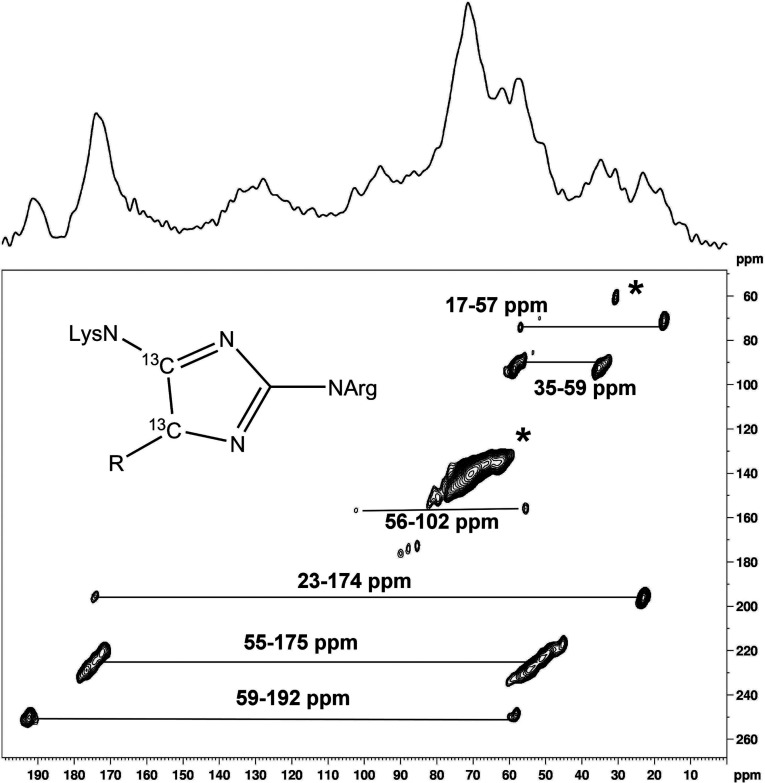
2D ssNMR characterization of [U-^13^C]ribose glycated collagen ^13^C ssNMR SQ–DQ (POST-C7) spectrum of [U-^13^C]ribose glycated collagen, in which correlations are seen only between carbon atoms directly bonded to each other. The output of this experiment is presented as a contour plot in which the horizontal axis corresponds to the 1D SQ–DQ spectrum (plotted above the 2D spectrum), and the vertical axis to a ‘double quantum’ dimension. In effect the horizontal chemical shift coordinate of each cross peak corresponds to the shift of one or other of the directly bonded carbons, and the vertical coordinate to the sum of both shifts. Relevant frequency pairings are indicated. Note that the correlation labelled ‘55–175 ppm’ corresponds to several correlations over a range of frequencies. Only species derived from the ^13^C_5_-ribose molecule will give signals in this spectrum. The inset depicts the imidazole lysine-arginine crosslink motif, which leads to a POST-C7 correlation between the carboxyimidamide carbon at ~175 ppm and the adjacent amino carbon at ~55 ppm. The possible AGEs with such a motif are DOPDIC (R=CH_2_CH(OH)CH_2_OH), which is derived directly from ribose, GODIC (R=H), derived from glyoxal and MODIC (R=Me), derived from methylglyoxal. Some signals (asterisked) correspond to ‘autocorrelations’ between signals from directly bonded carbons with very similar or identical chemical shifts. See Table 2 for assignments.

Incubation of collagen with [U-^13^C]ribose led to numerous reaction products derived from the ribose, whose NMR signals could be separated from those of the unaltered collagen components using double-quantum filtering in the NMR experiment (the 1D POST-C7 experiment; Figure 3). This procedure removes all signals from ^13^C atoms not directly bonded to another ^13^C so effectively only shows those which originate from [U-^13^C]ribose. Assignment was aided by 2D SQ–DQ correlations between covalently bonded ^13^C pairs (Figure 4), which demonstrated that many signals are superpositions of signals from two or more AGEs.

The most intense signal in the ^13^C-^13^C SQ–DQ spectrum is from secondary alcohols at 65–75 ppm, such as the Amadori product ribuloselysine and certain AGEs such as DOPDIC (N6-{2-{[(4S)-4-ammonio-5-oxido-5-oxopentyl]amino}-5-[(2S)-2,3-ydihydroxypropyl]-3,5-dihydro-4H-imidazol-4-ylidene}-L-lysinate) and glyceric acid. The strong correlation between 35 and 59 ppm is evidence for pentosinane and/or its hydrolysis product DOPDIC [11]. Correlations between 55 and 175 ppm are consistent with AGEs which share the imidazole lysine-arginine crosslink motif (Figure 4), the closely related pentosinane, and CML and CEL. The correlation between 17 and 57 ppm corresponds to a methyl group adjacent to an amine, functionalized to give in turn a correlation between 23 and 174 ppm, characteristic of an acetyl group in an acid, ester or amide. It is probably due to N^ε^-acetyl lysine, as the alternative, an ester, has never been reported as a stable AGE. The correlation between 59 and 192 ppm cannot be explained by any of the established AGEs, but is entirely consistent with the newly characterized norpronyl-lysine. The weakest correlation between 56 and 102 ppm corresponds to the furanose form of ribuloselysine [27]. Its persistence after 69 days of reaction suggests the Amadori product is more stable in the solid state than in solution. NMR spectral assignments thus far are summarized in Table 2. (Two autocorrelations, of the 31 ppm, and the 88 ppm, signals to themselves or other atoms with very similar shifts, have not yet been assigned.)

**Table 2:** A summary of the ^13^C POST-C_7_ correlations observed in collagen glycated by [U-^13^C]ribose over 69 days, with assignments

POST-C_7_ correlations	Assignment	
Signal 1/ppm	Signal 2/ppm	
17	57	MODIC and/or CEL
23	174	*N^ε^*-acetyl lysine or acetic acid
31	31	?
35	59	DOPDIC and/or pentosinane
45–60	170–180	DOPDIC, MODIC, GODIC, pentosinane, CML, CEL
56	102	ribuloselysine
59	192	‘norpronyl-lysine’
65–75	65–75	Any vicinal diol
88	88	?

^13^C NMR of the supernatant at the end of the incubation showed a variety of soluble AGEs, including large quantities of glycolic and oxalic acids (Supplementary Data Section S7 Figure S7, http://www.bioscirep.org/bsr/034/bsr034e096add.htm). In addition there were many new signals that did not correspond to anything seen in any of the model systems, particularly several signals between 20 and 40 ppm (Supplementary Data Section S7 Figure S8). Signals due to CML were observed, which suggests that some protein or amino acids have been released into solution.

The soluble AGEs produced from ribosylation of collagen bear limited resemblance to those produced by the PLL model. This shows that PLL does not completely mimic the Maillard reactions of collagen, and a more sophisticated model system is required to help determine the species present in the supernatant. In particular PLL does not replicate the considerable proportion of glycation-reactive arginyl residues in collagen (ca. 5% versus about 3.5% lysine in Type I collagen). It is also devoid of other amino acid functionalities, post-translational modifications and stable higher order structures (such as the collagen triple helices and fibril aggregates), any of which may exert unknown influences on glycation reactions, and increase glycation product diversity. It is interesting to note that very little of the acetic and formic acids that might have been expected as a result of the model systems are observed in the supernatant.

#### Collagen glycation by R5P

Under biomimetic reaction conditions, changes in collagen due to unlabelled R5P glycation were hard to detect by ssNMR beyond a general reduction in ^13^C linewidths. Thus in order to observe glycation products more easily, the reaction was facilitated through slightly more basic (pH 9) conditions and sonicating the collagen to disperse it and ensure as many reactive (lysyl, arginyl) residues as possible were accessible for reaction.

**Figure 5:**
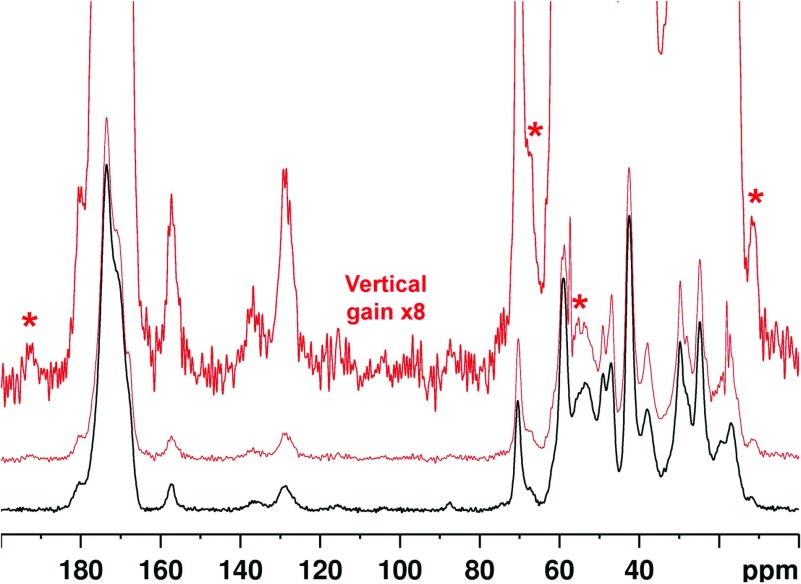
^13^C ssNMR characterization of collagen glycation by R5P ^13^C ssNMR spectrum of collagen after 14 days incubation (black, bottom trace), and collagen glycated by R5P for the same period after sonication and at pH9 (red, upper traces). Asterisked areas indicate where new signal appears from AGEs, identified in all cases by signal-to-noise ratios of greater than 2. The sharp signals at 18 and 58 ppm are due to ethanol present as an impurity in the R5P commercial starting material; that they persist after several washes implies the ethanol is closely bound into the collagen matrix.

The results of collagen glycation by R5P are shown in Figure 5. The slight sharpening of collagen signals is attributed to an increase in molecular hydration and molecular mobility after sonication and glycation. Areas where there appears to be new AGE signals are asterisked. The 12, 68 and 192 ppm signals are also present in PLL after R5P glycation. The prominence of 192 ppm suggests norpronyl-lysine is again present as an important AGE. The 12 ppm signal could be from norpronyl-lysine, MODIC, CEL or a combination thereof. The 68 ppm signal could be from any of the various AGEs containing an alcohol group. The 56 ppm signal is currently unassigned, and it is unclear whether this is a genuine AGE signal or a collagen signal which becomes apparent from the reduction in linewidths for the sonicated, glycated sample.

## DISCUSSION

NMR in both the liquid and solid phases is well known as a technique giving detailed chemical information on the composition of biological fluids and tissues in a near-native state. Characterization generally does not require separation of components, prior assumptions about possible composition, or that molecules of interest fortuitously possess distinctive spectroscopic ‘marker’ properties such as fluorescence. These factors have facilitated identification of a new glycation product arising from ribose and R5P glycation for which we propose the common name norpronyl lysine, and some previously unknown and unexpected aspects of biological glycation processes and products, such as amine catalysed sugar epimerizations under mild, approximately biological conditions.

Ribose [35], and especially R5P and ADPR [36–38], are vigorous glycating chemicals. Ribose and R5P are ubiquitous and key intermediates in several biochemical pathways including nucleotide biosynthesis and degradation and the pentose phosphate pathway, and can act as important carbon and energy sources. R5P attains significant levels greater than approximately millimolar in some tissues [39], certainly high enough to raise the possibility of its acting as a reactive glycating agent. Type 2 diabetes is characterized by activation of multiple inflammatory processes, mediators and pathways [40,41]; vascular damage leading to atherosclerosis is a typical pathological consequence [42] partly mediated in fact by specific RAGE (receptor of AGE) which initiate further cascades of inflammation [43].

ADPR and PAR (poly-ADP-ribose) are long recognized major components in crucial cell signalling, control and repair pathways [44], and there are intensive efforts to discover pharmacological modulators [45]. Potential benefits include down-regulation of multiple parallel pathways of inflammation and tissue injury in, for instance, diabetic complications. Important components of these pathways include enzymes such as PARG (PAR glycohydrolase) [46] and ADP-ribosyl protein lyase, which rapidly [44] generate free ADPR from PAR. This hydrolysis exposes the reactive ribosyl 1′′-anomeric carbons, which are protected by glycosidic conjugation in PAR. The reactive species, normally intracellular, will flood out of necrotizing cells and be able to react with extracellular matrix, which they will do much faster than the comparatively stable glucose.

In addition to the real possibility of these pentose sugars and their derivatives acting as significant glycating agents in their own right, the reactions they undergo with proteins reproduce mechanistically many if not all of the glycating actions of generally more abundant but much more sluggish reactants such as glucose and other hexoses and hexose phosphates. This not only makes the pentoses attractive reagents for understanding the nature and effects of the more abundant sugars, it raises the possibility of performing *in vitro* whole tissue or even *in vivo* experiments with labelled reactive glycating agents with subsequent *ex vivo* NMR and other, e.g. MS, analyses greatly facilitated by the resultant increase in sensitivity and discriminating power.

## Online data

Supplementary data

## References

[1] Huebschmann A. G., Regensteiner J. G., Vlassara H., Reusch J. E. B. (2006). Diabetes and advanced glycoxidation end products. Diab. Care.

[2] Monnier V. M., Sell D. R., Nagaraj R. H., Miyata S., Grandhee S., Odetti P., Ibrahim S. A. (1992). Maillard reaction-mediated molecular damage to extracellular-matrix and other tissue proteins in diabetes, aging, and uremia. Diabetes.

[3] Avery N. C., Bailey A. J. (2005). Enzymic and non-enzymic cross-linking mechanisms in relation to turnover of collagen: relevance to aging and exercise. Scand. J. Med. Sci. Sports.

[4] Sandwick R., Johanson M., Breuer E. (2005). Maillard reactions of ribose 5–phosphate and amino acids. Ann. N. Y. Acad. Sci..

[5] Munanairi A., O’Banion S. K., Gamble R., Breuer E., Harris A. W., Sandwick R. K. (2007). The multiple Maillard reactions of ribose and deoxyribose sugars and sugar phosphates. Carbohydr. Res..

[6] Wrobel K., Wrobel K., Garay-Sevilla M. E., Nava L. E., Malacara J. M. (1997). Novel analytical approach to monitoring advanced glycosylation end products in human serum with on-line spectrophotometric and spectrofluorometric detection in a flow system. Clin. Chem..

[7] Kume S., Takeya M., Mori T., Araki N., Suzuki H., Horiuchi S., Kodama T., Miyauchi Y., Takahashi K. (1995). Immunohistochemical and ultrastructural detection of advanced glycation end-products in atherosclerotic lesions of human aorta with a novel specific monoclonal-antibody. Am. J. Pathol..

[8] Vallejo-Cordoba B., Gonzalez-Cordova A. F. (2007). CE: A useful analytical tool for the characterization of Maillard reaction products in foods. Electrophoresis.

[9] Dunn J. A., Patrick J. S., Thorpe S. R., Baynes J. W. (1989). Oxidation of glycated proteins–age-dependent accumulation of N-epsilon-(carboxymethyl)lysine in lens proteins. Biochemistry.

[10] Sell D. R., Biemel K. M., Reihl O., Lederer M. O., Strauch C. M., Monnier V. M. (2005). Glucosepane is a major protein cross-link of the senescent human extracellular matrix–relationship with diabetes. J. Biol. Chem..

[11] Biemel K. M., Reihl O., Conrad J., Lederer M. O. (2001). Formation pathways for lysine-arginine cross-links derived from hexoses and pentoses by Maillard processes–unravelling the structure of a pentosidine precursor. J. Biol. Chem..

[12] Biemel K. M., Conrad J., Lederer M. O. (2002). Unexpected carbonyl mobility in aminoketoses: the key to major Maillard crosslinks. Angew. Chem. Int. Ed. Engl..

[13] Wells-Knecht K. J., Zyzak D. V., Litchfield J. E., Thorpe S. R., Baynes J. W. (1995). Mechanism of autoxidative glycosylation–identification of glyoxal and arabinose as intermediates in the autoxidative modification of proteins by glucose. Biochemistry.

[14] Wells-Knecht M. C., Thorpe S. R., Baynes J. W. (1995). Pathways of formation of glycoxidation products during glycation of collagen. Biochemistry.

[15] Ferreira A. E. N., Freire A. M. J. P., Voit E. O. (2003). A quantitative model of the generation of N-epsilon-(carboxymethyl)lysine in the Maillard reaction between collagen and glucose. Biochem. J..

[16] Chuyen N. V., Kurata T., Fujimaki M. (1973). Formation of N-carboxymethyl amino-acid from reaction of alpha-amino-acid with glyoxal. Agric. Biol. Chem..

[17] Smuda M., Voigt M., Glomb M. A. (2010). Degradation of 1–deoxy-D-erythro-hexo-2,3-diulose in the presence of lysine leads to formation of carboxylic acid amides. J. Agric. Food Chem..

[18] Henning C., Smuda M., Girndt M., Ulrich C., Glomb M. A. (2011). Molecular Basis of Maillard amide-advanced glycation end product (AGE) formation *in Vivo*. J. Biol. Chem..

[19] Monnier V. M., Mustata G. T., Biemel K. L., Reihl O., Lederer M. O., Dai Z. Y., Sell D. R. (2005). Cross-linking of the extracellular matrix by the Maillard reaction in aging and diabetes–An update on ‘a puzzle nearing resolution’. Ann. N. Y. Acad. Sci..

[20] Rizzi G. P. (2004). Role of phosphate and carboxylate ions in Maillard browning. J. Agric. Food Chem..

[21] Delatour T., Fenaille F., Parisod V., Vera F. A., Buetler T. (2006). Synthesis, tandem MS- and NMR-based characterization, and quantification of the carbon 13–labeled advanced glycation endproduct, 6–N-carboxymethyllysine. Amino Acids.

[22] Maekawa Y., Sugiura M., Takeuchi A., Tomoo K., Ishida T., Kamigauchi M. (2010). Study of lysozyme glycation reaction by mass spectrometry and NMR spectroscopy. Helvet. Chim. Acta.

[23] Howard M. J., Smales C. M. (2005). NMR analysis of synthetic human serum albumin alpha-helix 28 identifies structural distortion upon Amadori modification. J. Biol. Chem..

[24] Hohwy M., Jakobsen H. J., Eden M., Levitt M. H., Nielsen N. C. (1998). Broadband dipolar recoupling in the nuclear magnetic resonance of rotating solids: a compensated C7 pulse sequence. J. Chem. Phys..

[25] King-Morris M. J., Serianni A. S. (1987). C-13 NMR-studies of [1-C-13]aldoses-empirical rules correlating pyranose ring configuration and conformation with C-13 chemical-shifts and C-13-C-13 spin couplings. J. Am. Chem. Soc..

[26] Gleason W. B., Barker R. (1971). Evidence for a hydride shift in alkaline rearrangements of D-ribose. Can. J. Chem..

[27] Hellwig M., Henle T. (2010). Formyline, a new glycation compound from the reaction of lysine and 3-deoxypentosone. Eur. Food Res. Technol..

[28] Cistola D. P., Small D. M., Hamilton J. A. (1982). Ionization behavior of aqueous short-chain carboxylic-acids–a C-13 NMR-study. J. Lipid Res..

[29] Hagen R., Roberts J. D. (1969). Nuclear magnetic resonance spectroscopy. ^13^C spectra of aliphatic carboxylic acids and carboxylate anions. J. Am. Chem. Soc..

[30] Zou J., Guo Z. J., Parkinson J. A., Chen Y., Sadler P. J. (1999). Gold(III)-induced oxidation of glycine. Chem. Commun..

[31] Hauck T., Hubner Y., Bruhlmann F., Schwab W. (2003). Alternative pathway for the formation of 4,5–dihydroxy-2,3-pentanedione, the proposed precursor of 4-hydroxy-5-methyl-3(2H)-furanone as well as autoinducer-2, and its detection as natural constituent of tomato fruit. Biochim. Biophys. Acta–Gen. Subj..

[32] Panneerselvam J., Aranganathan S., Nalini N. (2009). Inhibitory effect of bread crust antioxidant pronyl-lysine on two different categories of colonic premalignant lesions induced by 1,2-dimethylhydrazine. Eur. J. Cancer Prev..

[33] Brinkmann E., Wells-Knecht K. J., Thorpe S. R., Baynes J. W. (1995). Characterization of an imidazolium compound formed by reaction of methylglyoxal and N-alpha-hippuryllysine. J. Chem. Soc., Perkin. Trans..

[34] Wells-Knecht K. J., Brinkmann E., Baynes J. W. (1995). Characterization of an imidazolium salt formed from glyoxal and N-alpha-hippuryllysine–a model for Maillard reaction cross-links in proteins. J. Org. Chem..

[35] Yan H., Harding J. J. (1997). Glycation-induced inactivation and loss of antigenicity of catalase and superoxide dismutase. Biochem. J..

[36] Cervantes-Laurean D., Jacobson E. L., Jacobson M. K. (1996). Glycation and glycoxidation of histones by ADP-ribose. J. Biol. Chem..

[37] Schreiber V., Dantzer F., Ame J. C., de Murcia G. (2006). Poly(ADP-ribose): novel functions for an old molecule. Nat. Rev. Mol. Cell Biol..

[38] Jacobson E. L., Cervantes-Laurean D., Jacobson M. K. (1994). Glycation of proteins by ADP-ribose. Mol. Cell. Biochem..

[39] Camici M., Tozzi M. G., Ipata P. L. (2006). Methods for the determination of intracellular levels of ribose phosphates. J. Biochem. Biophys. Methods.

[40] Donath M. Y., Shoelson S. E. (2011). Type 2 diabetes as an inflammatory disease. Nat. Rev. Immunol..

[41] Wellen K. E., Hotamisligil G. S. (2005). Inflammation, stress, and diabetes. J. Clin. Invest..

[42] Goldin A., Beckman J. A., Schmidt A. M., Creager M. A. (2006). Advanced glycation end products–sparking the development of diabetic vascular injury. Circulation.

[43] Park L., Raman K. G., Lee K. J., Lu Y., Ferran L. J., Chow W. S., Stern D., Schmidt A. M. (1998). Suppression of accelerated diabetic atherosclerosis by the soluble receptor for advanced glycation endproducts. Nat. Med..

[44] Juarez-Salinas H., Sims J. L., Jacobson M. K. (1979). Poly(ADP-Ribose) levels in carcinogen-treated cells. Nature.

[45] Jagtap P., Szabo C. (2005). Poly(ADP-ribose) polymerase and the therapeutic effects of its inhibitors. Nat. Rev. Drug Discov..

[46] Davidovic L., Vodenicharov M., Affar E. B., Poirier G. G. (2001). Importance of poly(ADP-ribose) glycohydrolase in the control of poly(ADP-ribose) metabolism. Exp. Cell Res..

