# Supplementary material for: A new glycation product ‘norpronyl-lysine,’ and direct characterization of cross linking and other glycation adducts: NMR of model compounds and collagen
Source: Biosci Rep. 2014 Mar 14;34(2):e00096. doi: 10.1042/BSR20130135 (PMC3953948; doi:10.1042/BSR20130135)
Supplement: Supplementary data [file bsr034e096add.pdf]

Biosci. Rep. (2014) / **34** / art:e00096 / doi 10.1042/BSR20130135

OPEN ACCESS

Original Paper

Bioscience Reports

SUPPLEMENTARY DATA

A new glycation product 'norpronyl-lysine,' and direct characterization of cross linking and other glycation adducts: NMR of model compounds and collagen

Peter T. B. BULLOCK*, David G. REID*, W. YING CHOW*, Wendy P. W. LAU* and Melinda J. DUER*[1]

*Department of Chemistry, University of Cambridge, Lensfield Road, Cambridge CB2 1EW, U.K.

www.bioscirep.org

Bioscience Reports

AGE formation

An ever growing number of AGEs have been identified and characterised in the last 20 years [1–6]. The many possible reaction pathways the initial Amadori product can explore is reflected in the structural variety of AGEs discovered so far (Figure S1).

Put broadly, the possible reaction routes fall into three groups, anticipated as early as 1953 [7].

The first category, that of products formed via formation of a reactive α-dicarbonyl species, is exemplified by pentosidine, a reaction product of pentose sugars [8]. This sort of α-dicarbonyl mechanism also leads to the formation of 4-hydroxyl-5-methyl-3(2H)-furanone (norfuraneol) from ribose as shown in Scheme S2 [9].

The second category involves fragmentation of the sugar carbon chain by retro-aldol reaction, hydrolytic cleavage or oxidative cleavage. The reactive sugar degradation products glyoxal and methylglyoxal in particular play a role in the formation of many AGEs. The two pathways to formation of CML [10,11] are a good example (Scheme S3), with oxidative cleavage of the Amadori product [3] competing with reaction of lysine with glyoxal [12].

Recently a third category of AGE has been discovered *in vitro* and *in vivo*: that of carboxylic amides, derived from β-dicarbonyl cleavage (Scheme S4) [13]. Of these N^{ε}-acetyl lysine and N^{ε}-formyl lysine are present in human plasma at comparable levels to CML [14].

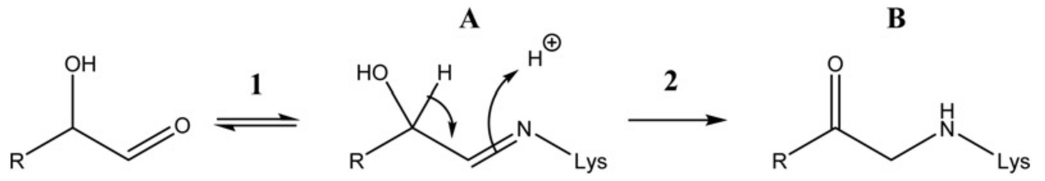

Scheme S1 The Maillard reaction occurs in three stages
Firstly, the sugar carbonyl group condenses with a terminal amine group (usually from a lysine residue) giving a Schiff base, (**A**) This subsequently undergoes an Amadori rearrangement yielding the more stable ketosamine (**B**). The ketosamine can then undergo a variety of transformations giving a plethora of AGEs.

[1] To whom correspondence should be addressed (email mjd13@cam.ac.uk).

© 2014 The Author(s) This is an Open Access article distributed under the terms of the Creative Commons Attribution Licence (CC-BY) (http://creativecommons.org/licenses/by/3.0/) which permits unrestricted use, distribution and reproduction in any medium, provided the original work is properly cited.

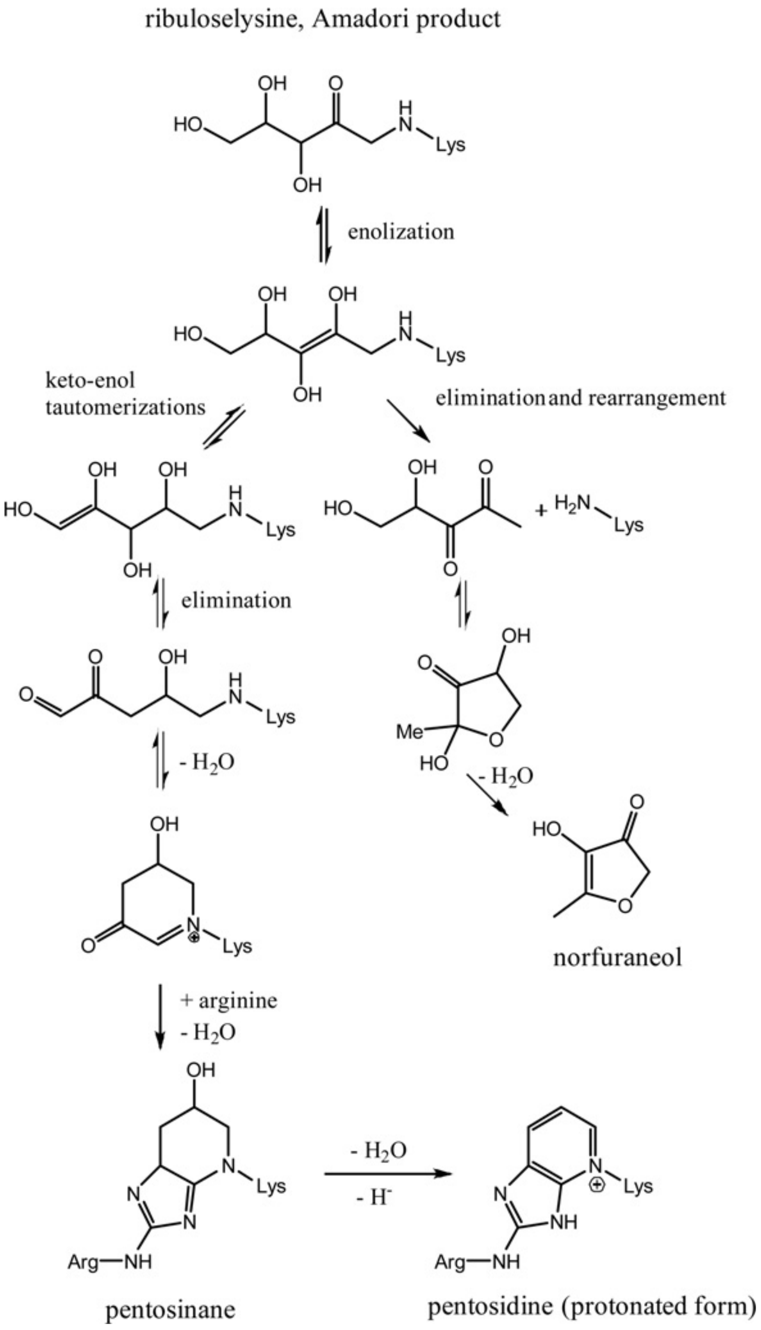

Scheme S2 **Mechanisms of formation of pentosinane, pentosidine and norfuraneol from ribuloselysine, the Amadori product of ribose and lysine**

© 2014 The Author(s) This is an Open Access article distributed under the terms of the Creative Commons Attribution Licence (CC-BY) (http://creativecommons.org/licenses/by/3.0/) which permits unrestricted use, distribution and reproduction in any medium, provided the original work is properly cited.

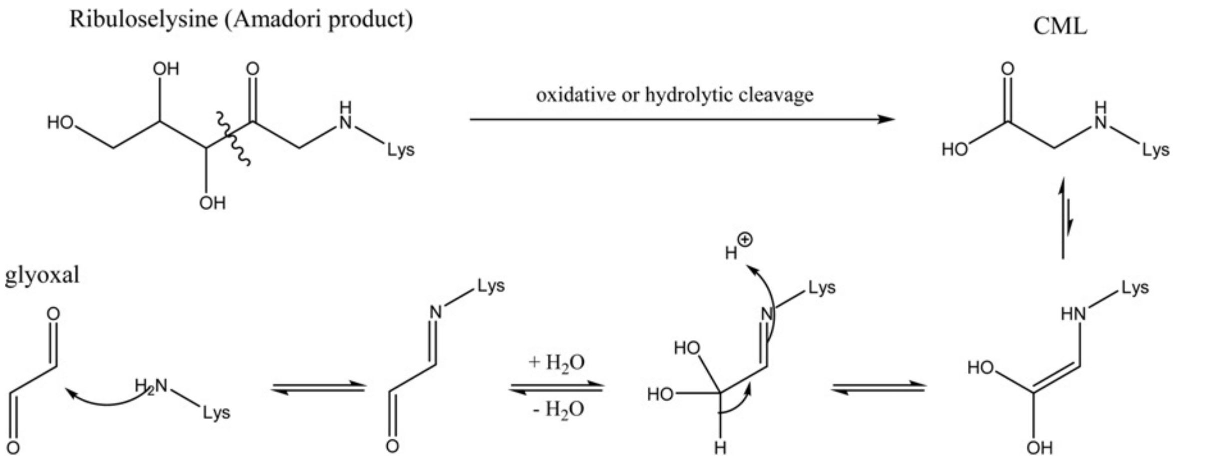

Scheme S3 **Mechanisms of formation of CML**
The pathway which dominates has been shown to depend on phosphate concentration and sugar concentration, with the glyoxal route dominant except at low phosphate and high sugar [10,11].

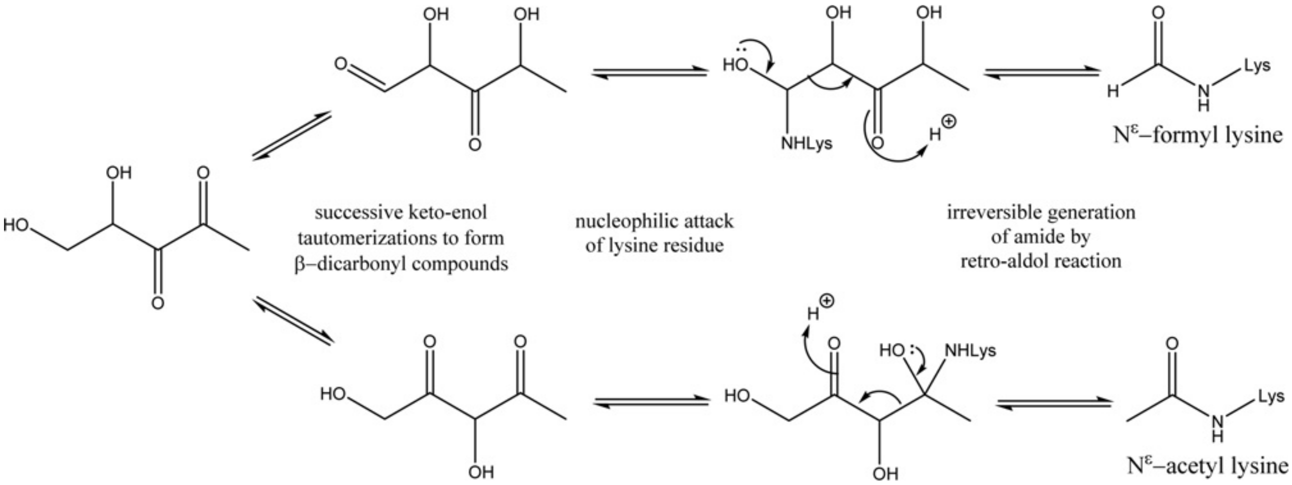

Scheme S4 **Mechanism of formation of N$^{\varepsilon}$-acetyl lysine and N$^{\varepsilon}$-formyl lysine from ribose**
The shown starting material is a degradation product of ribuloselysine (Scheme S2). N$^{\varepsilon}$-lactoyl lysine and N$^{\varepsilon}$-glycolyl lysine can also be formed if the lysine residue attacks the other carbonyl group in the β-dicarbonyl intermediate.

© 2014 The Author(s) This is an Open Access article distributed under the terms of the Creative Commons Attribution Licence (CC-BY) (http://creativecommons.org/licenses/by/3.0/) which permits unrestricted use, distribution and reproduction in any medium, provided the original work is properly cited.

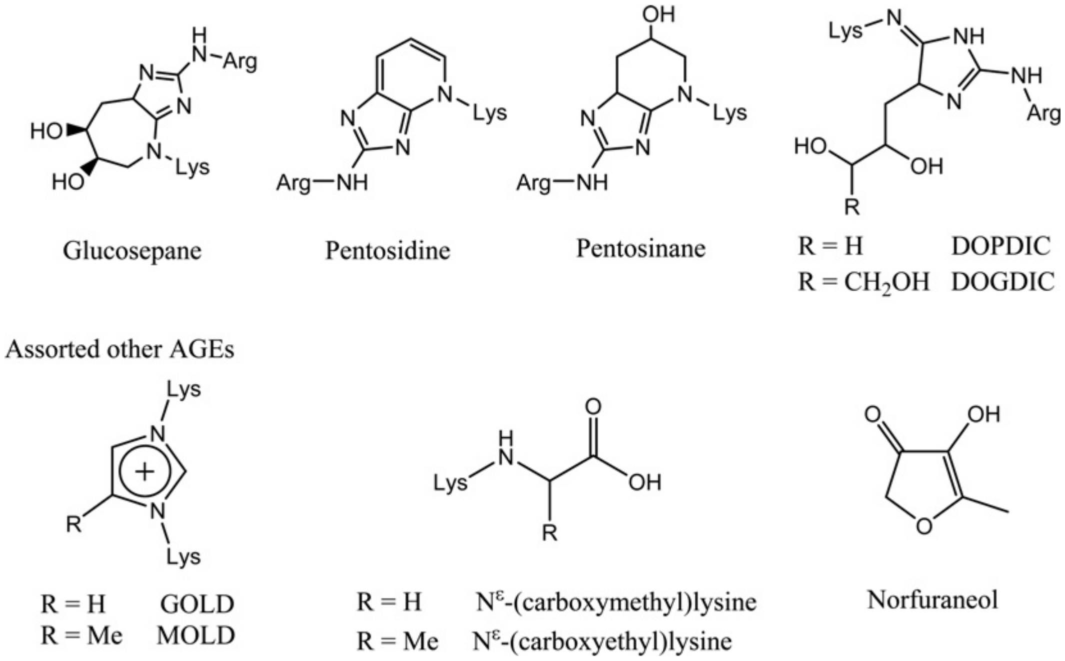

Figure S1 Some important AGEs
Glucosepane is considered the most important known crosslink [10], being derived from glucose. Pentosidine and its precursor pentosinane are formed in the same manner as glucosepane from pentose sugars (which are formed by the autoxidation of glucose). **DOGDIC** (N6-{2-{[(4S)-4-ammonio-5-oxido-5-oxopentyl]amino}-5-[(2S,3R)-2,3,4-trihydroxybutyl]-3,5-dihydro-4H-imidazol-4-ylidene}-L-lysinate) and **DOPDIC** are hydrolysed forms of glucosepane and pentosinane, respectively. **GOLD** and N^{ε}-**CML** are the products formed by reaction of lysine with glyoxal (a product from the oxidation of sugars). MOLD (methylglyoxal lysine dimer) and N^{ε}-**CEL** are the analogous products formed from methylglyoxal (a product from the fragmentation of sugars). Norfuraneol is formed from the amine-catalysed rearrangement and dehydration of pentose sugars.

© 2014 The Author(s) This is an Open Access article distributed under the terms of the Creative Commons Attribution Licence (CC-BY) (http://creativecommons.org/licenses/by/3.0/) which permits unrestricted use, distribution and reproduction in any medium, provided the original work is properly cited.

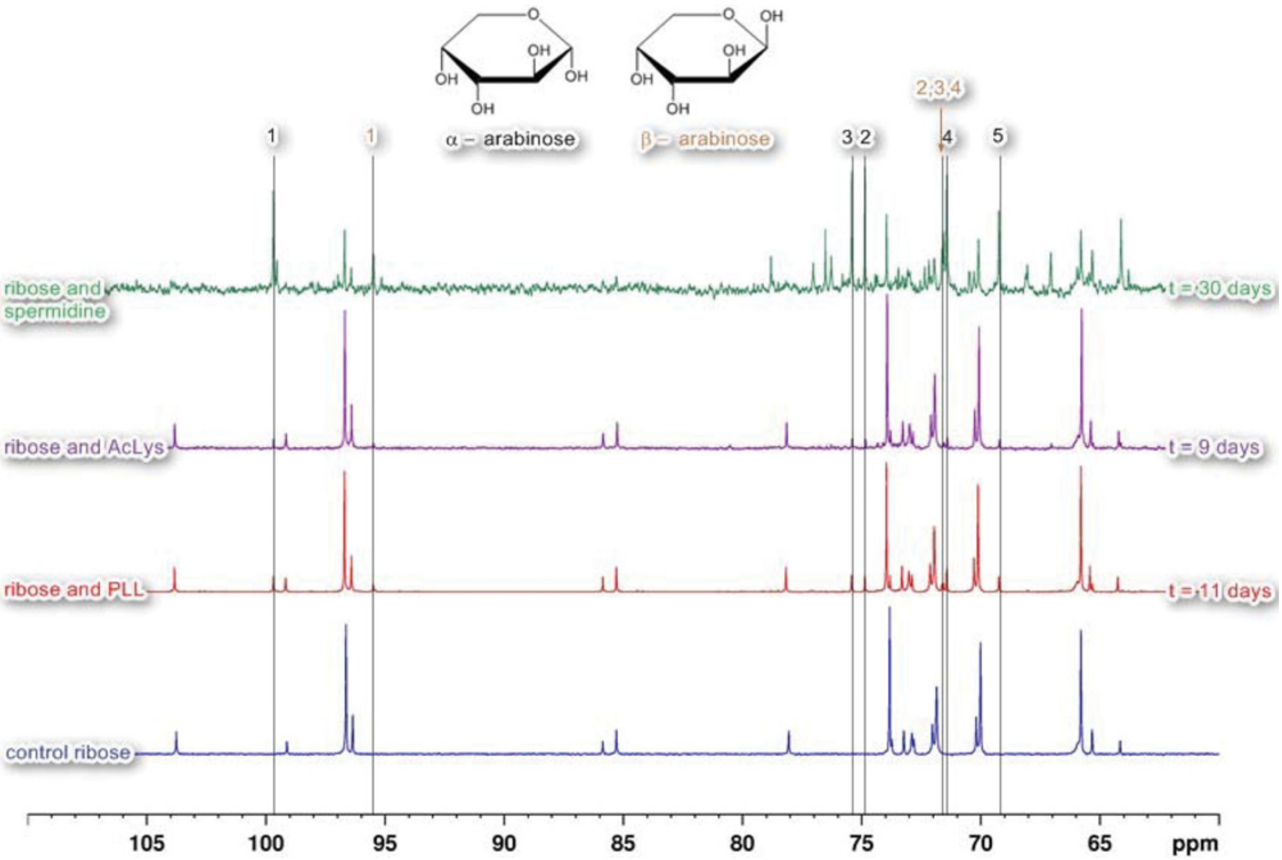

Figure S2 **^{13}C solution-state NMR spectra of incubations of ribose with (a) spermidine after 30 days (green), (b) AcLys after 9 days (purple), (c) PLL after 11 days (red) and (d) no amine present (blue)**
In each case the peaks at δ69.2, 71.5 (multiple), 74.8, 75.4, 95.5 and 99.7 ppm which are not present in a control incubation of ribose in the absence of amine are diagnostic of formation of arabinose, the C2-epimer of ribose,with the assignments given.

© 2014 The Author(s) This is an Open Access article distributed under the terms of the Creative Commons Attribution Licence (CC-BY) (http://creativecommons.org/licenses/by/3.0/) which permits unrestricted use, distribution and reproduction in any medium, provided the original work is properly cited.

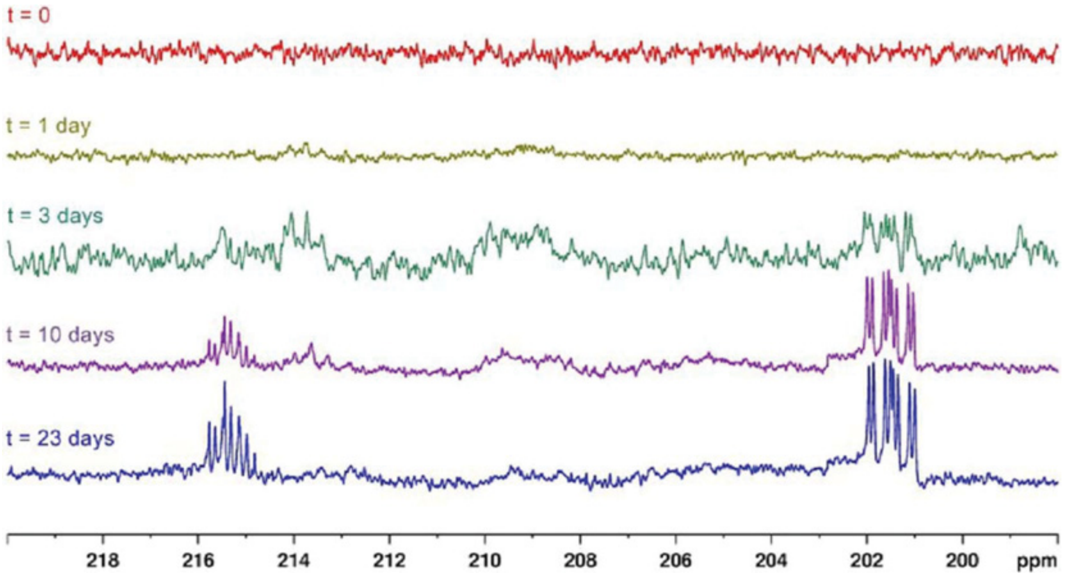

Figure S3 **^{13}C solution-state NMR spectra of the reaction between [U-^{13}C]ribose and PLL with data taken over a period of 23 days**

The broad signal at 209 ppm, assigned to the carbonyl carbon of ribuloselysine, the Amadori product, reaches a maximum after 3 days before slowly decaying, although it is still present after 23 days suggesting the glycation process had not yet gone to completion. The broadness of the signal is presumably attributable to the polymeric nature of PLL: each ribuloselysine entity is in a slightly different chemical environment. The multiplet (doublet of doublets of doublet) at 202 ppm couples to 136 ppm and is from norfuraneol. The multiplet around 215 ppm is from relatively stable ketone intermediates; a corresponding feature is also present in reactions of R5P.

© 2014 The Author(s) This is an Open Access article distributed under the terms of the Creative Commons Attribution Licence (CC-BY) (http://creativecommons.org/licenses/by/3.0/) which permits unrestricted use, distribution and reproduction in any medium, provided the original work is properly cited.

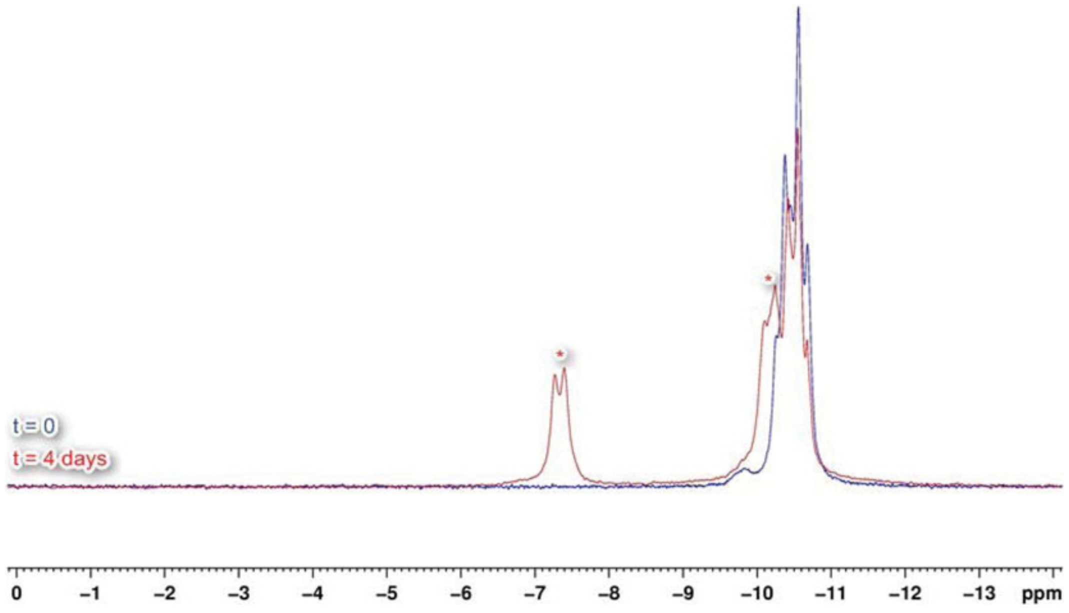

Figure S4 **^{31}P solution-state NMR of ADPR before (blue) and after (red) reaction with PLL**
The ADPR signal loses intensity and is replaced with two doublets (asterisked) at -7.3 and -10.2 ppm ($J = 20$ Hz) which correspond to the β- and α-phosphate groups of free ADP, released from ADPR, respectively.

© 2014 The Author(s) This is an Open Access article distributed under the terms of the Creative Commons Attribution Licence (CC-BY) (http://creativecommons.org/licenses/by/3.0/) which permits unrestricted use, distribution and reproduction in any medium, provided the original work is properly cited.

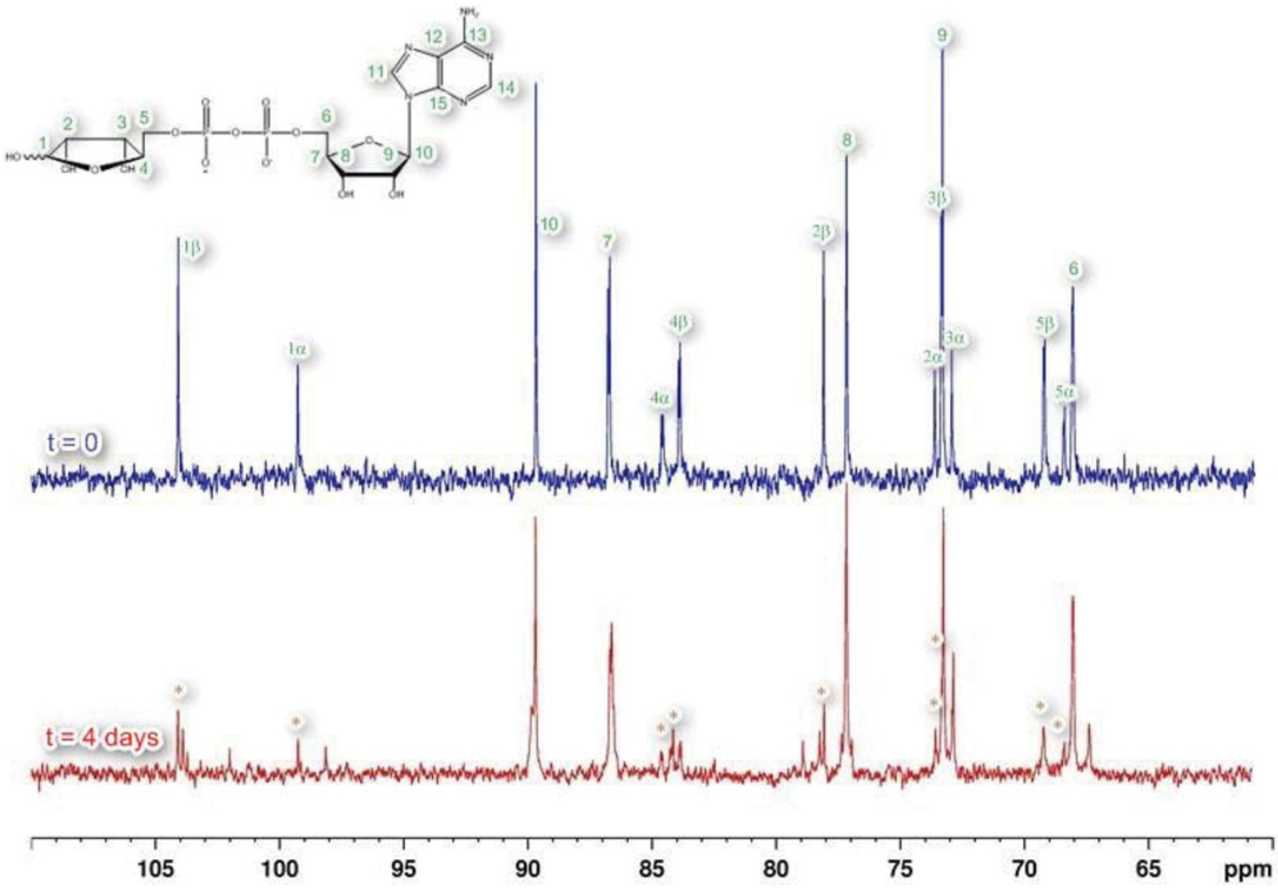

Figure S5 **^{13}C solution-state NMR of ADPR before (blue) and after (red) reaction with PLL**
ADPR assignment is given. The chemical shifts of C_1–C_5 of the ribosyl phosphate moiety are dependent on the configuration of the anomeric carbon, C_1, and accordingly signals from the α-anomer (hydroxyl group 'down') and the β-anomer (hydroxyl group 'up') are differentiated. After the generation of a precipitate (see text for details), the signals from the free ribose, asterisked, are significantly reduced, whereas the signals from ADP are unchanged in intensity. Taken with Figure S4, these results confirm that the ADP is released during the reaction that leads to crosslinking and precipitation. The same signal depletion over time was observed for glycation of PLL by R5P.

© 2014 The Author(s) This is an Open Access article distributed under the terms of the Creative Commons Attribution Licence (CC-BY) (http://creativecommons.org/licenses/by/3.0/) which permits unrestricted use, distribution and reproduction in any medium, provided the original work is properly cited.

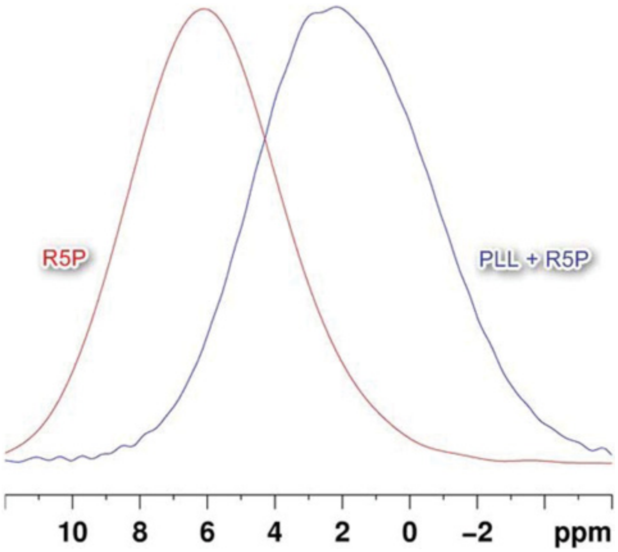

Figure S6 ^{31}P ssNMR of R5P before (red) and after (blue) reaction with PLL

The ^{31}P chemical shift changes from the phosphomonoester shift of 6.1–2.3 ppm of free phosphate ion. This further demonstrates that phosphate acts as a leaving group in the Maillard reactions of R5P.

© 2014 The Author(s) This is an Open Access article distributed under the terms of the Creative Commons Attribution Licence (CC-BY) (http://creativecommons.org/licenses/by/3.0/) which permits unrestricted use, distribution and reproduction in any medium, provided the original work is properly cited.

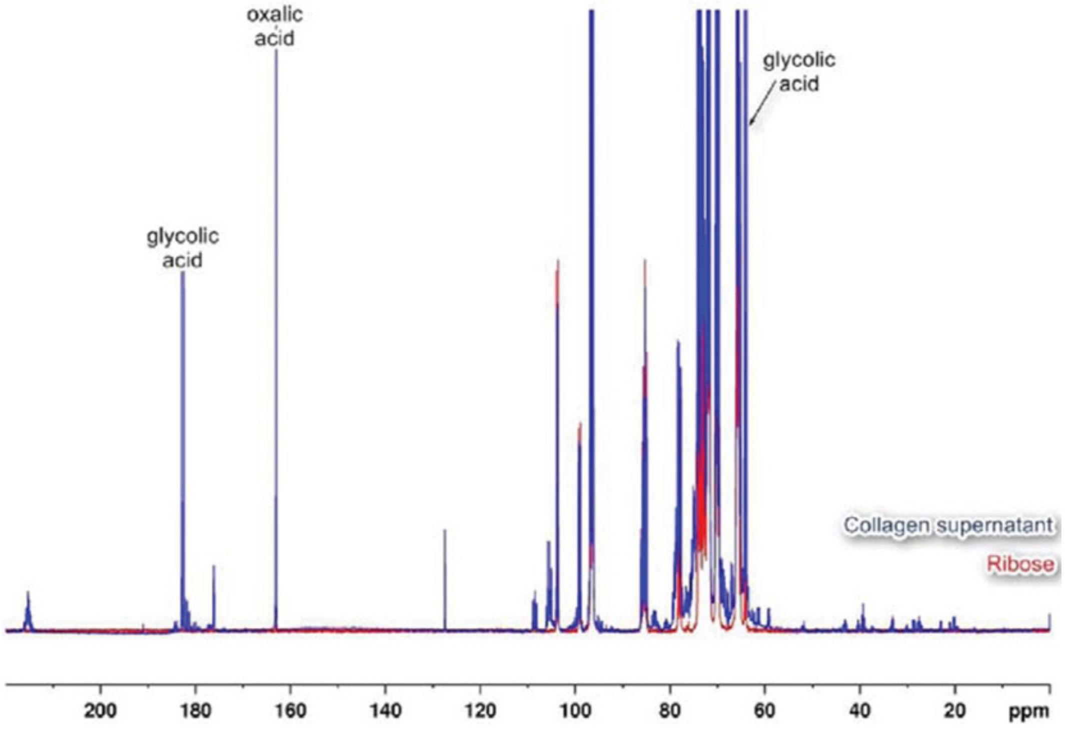

Figure S7 **^{13}C solution-state NMR spectrum of the supernatant phosphate buffer from [U-^{13}C]ribose glycated collagen (blue) and that of unreacted [U-^{13}C]ribose (red)**
The dominant species in the supernatant is unreacted ribose, but significant quantities of glycolic and oxalic acids were also observed (signals indicated, see Section 5.1.2), along with a multitude of weaker signals from minor AGEs which require further assignment.

© 2014 The Author(s) This is an Open Access article distributed under the terms of the Creative Commons Attribution Licence (CC-BY) (http://creativecommons.org/licenses/by/3.0/) which permits unrestricted use, distribution and reproduction in any medium, provided the original work is properly cited.

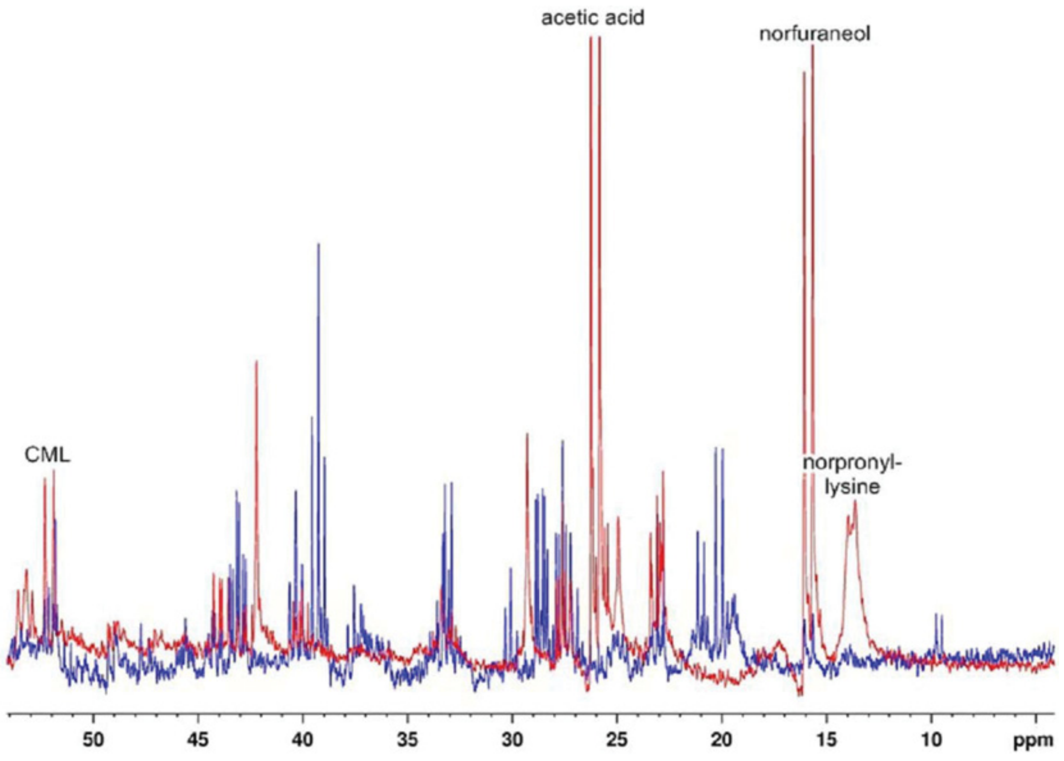

Figure S8 **Comparison of the ^{13}C NMR spectrum of the supernatant from [U-^{13}C]ribose glycated collagen (blue) and the model system: [U-^{13}C]ribose glycated PLL (red)**
Several weak signals are observed in both spectra, such the signal due to CML at 52 ppm. However, many of the supernatant AGE signals are not observed in the model system, showing that PLL is an incomplete model for collagen glycation. The lack of norpronyl-lysine in the supernatant is expected as it is attached to the solid collagen. The absence of norfuraneol and acetic acid is surprising: either they are not generated in observable quantities, or bind strongly to collagen, or had degraded by the time the spectrum was obtained.

Table S1 Shifts of C_1 and C_2 in carboxylic acids are highly pH and concentration dependent, with carboxylate ion C_1 typically resonating 4–5 ppm to higher frequency than the corresponding acid [15]
Values in the table are literature values for the acid form [16–18]. Formate resonated at 173.8 ppm, acetate at 184.1 and 26.1 ppm, glycolate at 182.6 and 62.1 ppm, oxalate at 163.0 ppm. Glyoxylate was not observed. These assignments were confirmed by peak multiplicity, *J* values, and ^{13}C–^{13}C COSY correlations, in isotopically enriched experiments.

	C_1 (ppm)	C_2 (ppm)
Formic acid	167	–
Acetic acid	178	22
Glycolic acid	177	60
Glyoxylic acid	174	90
Oxalic acid	161	161

© 2014 The Author(s) This is an Open Access article distributed under the terms of the Creative Commons Attribution Licence (CC-BY) (http://creativecommons.org/licenses/by/3.0/) which permits unrestricted use, distribution and reproduction in any medium, provided the original work is properly cited.

Table S2 A summary of the carboxylic acids generated in the Maillard reactions between model amines and sugars

A tick indicates that the species is appreciably present, a tick in parentheses that it is weak, a cross that there is no evidence of the species.

Reactants	Formic acid	Acetic acid	Glycolic acid	Glyoxylic acid	Oxalic acid
Ribose and PLL	√?	×	√	×	×
[U-^{13}C]-ribose and PLL	√	√	√	×	(√)
R5P and PLL	(√)	×	√	×	×
[U-^{13}C]-ribose and collagen	(√)	(√)	√	×	√
Ribose and AcLys	√	√	√	×	×
[U-^{13}C]-ribose and AcLys	√	√	√	×	(√)
R5P and AcLys	√	√	×	×	×
Ribose and spermidine	√	√	√	×	×

Table S3 Full ^{13}C solution-state NMR assignment of $^{13}C_5$-norfuraneol as determined from its observation as a product of the reaction between [U-^{13}C]ribose and PLL

The assignment of C_5 was aided by the detection of norfuraneol as a product of reaction between ribose and spermidine, where its NMR signal did not overlap with others.

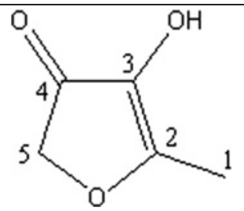

Carbon atom	δ (ppm)	Multiplicity	J (Hz)
1	15.9	D	49
2	183.5	Ddd	88, 49, 14
3	136.1	Dddd	88, 65, 20, 3
4	201.5	Ddd	65, 43, 14
5	76.5	Dd	43, 20

© 2014 The Author(s) This is an Open Access article distributed under the terms of the Creative Commons Attribution Licence (CC-BY) (http://creativecommons.org/licenses/by/3.0/) which permits unrestricted use, distribution and reproduction in any medium, provided the original work is properly cited.

REFERENCES

1 Wells-Knecht, K. J., Brinkmann, E., WellsKnecht, M. C., Litchfield, J. E., Ahmed, M. U., Reddy, S., Zyzak, D. V., Thorpe, S. R. and Baynes, J. W. (1996) New biomarkers of Maillard reaction damage to proteins. Nephrol. Dialy Transplant. **11 (Suppl. 5)**, 41–47

2 Biemel, K. M., Reihl, O., Conrad, J. and Lederer, M. O. (2001) Formation pathways for lysine-arginine cross-links derived from hexoses and pentoses by Maillard processes-unraveling the structure of a pentosidine precursor. J. Biol. Chem. **276**, 23405–23412

3 Dunn, J. A., Patrick, J. S., Thorpe, S. R. and Baynes, J. W. (1989) Oxidation of glycated proteins – age-dependent accumulation of N-epsilon-(carboxymethyl)lysine in lens proteins. Biochemistry **28**, 9464–9468

4 Monnier, V. M., Mustata, G. T., Biemel, K. L., Reihl, O., Lederer, M. O., Dai, Z. Y. and Sell, D. R. (2005) Cross-linking of the extracellular matrix by the Maillard reaction in aging and diabetes-an update on “a puzzle nearing resolution”. Ann. N. Y. Acad. Sci. **1043**, 533–544

5 Munanairi, A., O’Banion, S. K., Gamble, R., Breuer, E., Harris, A. W. and Sandwick, R. K. (2007) The multiple Maillard reactions of ribose and deoxyribose sugars and sugar phosphates. Carbohydr. Res. **342**, 2575–2592

6 Biemel, K. M., Friedl, D. A. and Lederer, M. O. (2002) Identification and quantification of major Maillard cross-links in human serum albumin and lens protein-evidence for glucosepane as the dominant compound. J. Biol. Chem. **277**, 24907–24915

7 Hodge, J. E. (1953) Dehydrated foods: chemistry of browning reactions in model systems. Agricult. Food Chem. **1**, 928–943

8 Biemel, K. M., Conrad, J. and Lederer, M. O. (2002) Unexpected carbonyl mobility in aminoketoses: the key to major Maillard crosslinks. Angew. Chemie. Int. Ed. Eng. **41**, 801–803

9 Hauck, T., Hubner, Y., Bruhlmann, F. and Schwab, W. (2003) Alternative pathway for the formation of 4,5-dihydroxy-2,3-pentanedione, the proposed precursor of 4-hydroxy-5-methyl-3(2H)-furanone as well as autoinducer-2, and its detection as natural constituent of tomato fruit. Biochim. Biophys. Acta-Gen. Subj. **1623**, 109–119

10 Ferreira, A. E. N., Freire, A. M. J. P. and Voit, E. O. (2003) A quantitative model of the generation of N-epsilon-(carboxymethyl)lysine in the Maillard reaction between collagen and glucose. Biochem. J. **376**, 109–121

11 Wells-Knecht, M. C., Thorpe, S. R. and Baynes, J. W. (1995) Pathways of formation of glycoxidation products during glycation of collagen. Biochem. **34**, 15134–15141

12 Chuyen, N. V., Kurata, T. and Fujimaki, M. (1973) Formation of N-carboxymethyl amino-acid from reaction of alpha-amino-acid with glyoxal. Agric. Biol. Chem. **37**, 2209–2210

13 Smuda, M., Voigt, M. and Glomb, M. A. (2010) Degradation of 1-deoxy-D-erythro-hexo-2,3-diulose in the presence of lysine leads to formation of carboxylic acid amides. J. Agric. Food Chem. **58**, 6458–6464

14 Henning, C., Smuda, M., Girndt, M., Ulrich, C. and Glomb, M. A. (2011) Molecular basis of maillard amide-advanced glycation end product (AGE) formation *in vivo*. J. Biol. Chem. **286**, 44350–44356

15 Cistola, D. P., Small, D. M. and Hamilton, J. A. (1982) Ionization behavior of queous Short-Chain Carboxylic-Acids-a C-13 NMR-Study. J. Lipid Res. **23**, 795–799

16 Hagen, R. and Roberts, J. D. (1969) Nuclear magnetic resonance spectroscopy. 13C spectra of aliphatic carboxylic acids and carboxylate anions. J. Am. Chem. Soc. **91**, 4504–4506

17 Zou, J., Guo, Z. J., Parkinson, J. A., Chen, Y. and Sadler, P. J. (1999) Gold(III)-induced oxidation of glycine. Chem. Commun. 1359–1360

18 Kalinowski, H.-O., Berger, S. and Braun, S. (1988) Carbon 13 NMR Spectroscopy, Wiley

Received 11 December 2013/15 January 2014; accepted 29 January 2014

Published as Immediate Publication 12 February 2014, doi 10.1042/BSR20130135

© 2014 The Author(s) This is an Open Access article distributed under the terms of the Creative Commons Attribution Licence (CC-BY) (http://creativecommons.org/licenses/by/3.0/) which permits unrestricted use, distribution and reproduction in any medium, provided the original work is properly cited.
